# Supplementary material for: Genome-wide dynamics of Pol II elongation and its interplay with promoter proximal pausing, chromatin, and exons
Source: eLife. 2014 Apr 29;3:e02407. doi: 10.7554/eLife.02407 (PMC4001325; doi:10.7554/eLife.02407)
Supplement: Figure 1—source data 1. — The top tables depict the total reads sequenced, and the alignment to the spike-in controls, ribosomal, reference genomes for each replicate. One of the 25 min Trp replicates was of poor quality due to RNA degradation during the library preparation and wasn’t included in further analysis. DOI: http://dx.doi.org/10.7554/eLife.02407.006 [file elife02407s001.docx]

Figure 1—source data 1

| **Sequencing results** | **Total reads** | **% trimmed** | **% ribosomal** | **aligned** | **% aligned** | **Spike-In** | **%**  **Spike-In** |
| --- | --- | --- | --- | --- | --- | --- | --- |
| **Control Trp #1** | 39054383 | 23.3% | 29.5% | 5016163 | 12.8% | 219906 | 0.6% |
| **12.5min Trp #1** | 35515502 | 24.6% | 24.3% | 5152395 | 14.5% | 338370 | 1.0% |
| **25min Trp #1** | 34107998 | 51.2% | 13.9% | 613603 | 1.8% | 73777 | 0.2% |
| **50min Trp #1** | 37503054 | 22.3% | 40.0% | 1824854 | 4.9% | 253943 | 0.7% |
| **Control Trp #2** | 74380410 | 20.3% | 31.7% | 13074860 | 17.6% | 393978 | 0.5% |
| **12.5min Trp #2** | 45737802 | 20.4% | 25.1% | 9147442 | 20.0% | 358560 | 0.8% |
| **25min Trp #2** | 76937683 | 24.0% | 33.2% | 8798933 | 11.4% | 805210 | 1.0% |
| **50min Trp #2** | 40672137 | 21.0% | 39.7% | 2639097 | 6.5% | 304272 | 0.7% |
| **untreated FP #1** | 104889824 | 16.2% | 34.8% | 25539711 | 24.3% | 3046032 | 2.9% |
| **2min FP #1** | 92103568 | 16.1% | 34.7% | 22843152 | 24.8% | 2627811 | 2.9% |
| **5min FP #1** | 82094962 | 16.7% | 30.0% | 24548283 | 29.9% | 2454930 | 3.0% |
| **12.5min FP #1** | 84600449 | 15.3% | 34.2% | 21607659 | 25.5% | 2980280 | 3.5% |
| **25min FP #1** | 94235237 | 16.7% | 41.1% | 17356268 | 18.4% | 3055098 | 3.2% |
| **50min FP #1** | 101614542 | 14.0% | 49.1% | 15403548 | 15.2% | 2721538 | 2.7% |
| **untreated FP #2** | 63997320 | 15.4% | 33.5% | 17869848 | 27.9% | 1295280 | 2.0% |
| **2min FP #2** | 41265136 | 12.4% | 35.5% | 12447634 | 30.2% | 901113 | 2.2% |
| **5min FP #2** | 50758538 | 14.9% | 36.9% | 12912212 | 25.4% | 1286001 | 2.5% |
| **12.5min FP #2** | 54675137 | 10.4% | 35.4% | 16926587 | 31.0% | 1873970 | 3.4% |
| **25min FP #2** | 74393287 | 9.6% | 48.6% | 15992442 | 21.5% | 2544132 | 3.4% |
| **50min FP #2** | 83154700 | 9.2% | 55.2% | 14019123 | 16.9% | 2124287 | 2.6% |
