# Supplementary material for: Genome-wide dynamics of Pol II elongation and its interplay with promoter proximal pausing, chromatin, and exons
Source: eLife. 2014 Apr 29;3:e02407. doi: 10.7554/eLife.02407 (PMC4001325; doi:10.7554/eLife.02407)
Supplement: Figure 1—source data 3. — Spearman correlations between the replicates for each time course after drug treatment in either the gene body or promoter regions. DOI: http://dx.doi.org/10.7554/eLife.02407.008 [file elife02407s003.docx]

Figure 1—source data 3

| **Spearman correlation** | **promoter** | **gene body** |
| --- | --- | --- |
| **Control Trp replicates** | 0.958 | 0.986 |
| **12.5min Trp replicates** | 0.937 | 0.988 |
| **25min Trp replicates** | - | - |
| **50min Trp replicates** | 0.772 | 0.964 |
| **untreated FP replicates** | 0.976 | 0.993 |
| **2min FP replicates** | 0.975 | 0.991 |
| **5min FP replicates** | 0.973 | 0.990 |
| **12.5min FP replicates** | 0.977 | 0.990 |
| **25min FP replicates** | 0.976 | 0.986 |
| **50min FP replicates** | 0.971 | 0.980 |
