# Supplementary material for: Genome-wide dynamics of Pol II elongation and its interplay with promoter proximal pausing, chromatin, and exons
Source: eLife. 2014 Apr 29;3:e02407. doi: 10.7554/eLife.02407 (PMC4001325; doi:10.7554/eLife.02407)
Supplement: Figure 2—source data 1. — The gene body change was measured in the region from 1 to 3.5 kb downstream of the TSS for each timepoint, and was called significantly increased when the read density ratio was greater then 1 with a Fisher Exact p-value <0.05, or significantly decreased when the read density ratio was smaller then 1 with a Fisher Exact p-value <0.05. The same for the pause peak increase and decrease, but using a 250 bp region with maximal read density in a window of ±500 bp around the annotated TSS. The divergent peak was found by using a 250 bp region with maximal read density in a window of −1000 to +500 bp around the annotated TSS on the antisense strand, and change was defined similarly as the gene body change. Pausing indexes (PI) are the ratio between gene body and promoter proximal Pol II density, and the change in PI has therefore no Fisher Exact p-value associated with it. PI’s are increased or decreased when the ratio between untreated and treated PI’s is greater or smaller then 1, respectively. DOI: http://dx.doi.org/10.7554/eLife.02407.011 [file elife02407s004.docx]

Figure 2—source data 1

| **Changed genes**  **(p < 0.05)** | **12.5min Trp** | **25min Trp** | **50min Trp** | **2min FP** | **5min FP** | **12.5min FP** | **25min FP** | **50min FP** |
| --- | --- | --- | --- | --- | --- | --- | --- | --- |
| **gene body decrease** | 4013 (62.9%) | 5849 (91.7%) | 6101 (95.6%) | 4065 (63.7%) | 5425 (85.0%) | 6047 (94.8%) | 6118 (95.9%) | 6071 (95.2%) |
| **gene body increase** | 178 (2.8%) | 28 (0.4%) | 7  (0.1%) | 270 (4.2%) | 25 (0.4%) | 44 (0.7%) | 40 (0.6%) | 85 (1.3%) |
| **pause peak decrease** | 4684 (73.4%) | 5500 (86.2%) | 5689 (89.2%) | 1948 (30.5%) | 1726 (27.1%) | 1377 (21.6%) | 1353 (21.2%) | 1161 (18.2%) |
| **pause peak increase** | 23  (0.4%) | 8 (0.1%) | 5  (0.1%) | 499 (7.8%) | 373 (5.8%) | 1787 (28.0%) | 3058 (47.9%) | 3601 (56.4%) |
| **divergent peak decrease** | 3733 (58.5%) | 4735 (74.2%) | 4914 (77.0%) | 1373 (21.5%) | 1512 (23.7%) | 1358 (21.3%) | 1848 (29.0%) | 1629 (25.5%) |
| **divergent peak increase** | 13  (0.2%) | 9 (0.1%) | 9  (0.1%) | 524 (8.2%) | 289 (4.5%) | 1234 (19.3%) | 2042 (32.3%) | 2670 (41.8%) |
| **PI decrease**  **(no p-value)** | 5162 (80.9%) | 4896 (76.7%) | 4982 (78.1%) | 2059 (32.3%) | 1174 (18.4%) | 431 (6.8%) | 359 (5.6%) | 320 (5.0%) |
| **PI increase**  **(no p-value)** | 1218 (19.1%) | 1484 (23.3%) | 1398 (21.9%) | 4321 (67.7%) | 5206 (81.6%) | 5949 (93.2%) | 6021 (94.4%) | 6060 (95.0%) |
