# Supplementary material for: Genome-wide dynamics of Pol II elongation and its interplay with promoter proximal pausing, chromatin, and exons
Source: eLife. 2014 Apr 29;3:e02407. doi: 10.7554/eLife.02407 (PMC4001325; doi:10.7554/eLife.02407)
Supplement: Figure 5—source data 1. — Complete table of all the ChIP-seq data and references taken into account in the correlation study between mid elongation rates (n = 938) and promoter or gene body occupancy of each of the listed factors. The references to the GEO datasets are shown in the “Major datasets” section. DOI: http://dx.doi.org/10.7554/eLife.02407.019 [file elife02407s006.docx]

Figure 5—source data 1

| **ChIP-seq datasets** | **Reference** |
| --- | --- |
| Pol II-Ser2, Pol II-Ser5, Pol II-60’ DMSO, Pol II-60’ FP, NelfA, Ctr9, Spt5 | Rahl et al., 2010 |
| Oct4, Sox2, Nanog, Tcf3, Suz12, H3K4me3, H3K36me3, H3K79me2 | Marson et al., 2008 |
| H3K4me1, H3K27Ac | Creyghton, 2010 |
| c-Myc, n-Myc, Oct4, Klf4, Nanog, Sox2, Esrrb, p300, CTCF, Smad1, Tcfcp2l1, E2f1, Zfx | Chen et al., 2008 |
| Cdk9, Ell2, Aff4 | Lin et al., 2011 |
| Ell | Smith et al., 2011 |
| Ezh2, Suz12, Ring1B | Ku et al., 2008 |
| Pol II, H4K4me3, H3K27me3, H3K36me3, H3K9me3, H4K20me3 | Mikkelsen et al., 2007 |
| H3K4me1, H3K4me2 | Meissner et al., 2008 |
| Smc1, Smc3, Med12, Med1, Nipbl | Kagey et al., 2010 |
| TBP, Taf1, Taf3, Pol II-unphosphorylated CTD | Liu et al., 2011 |
| Chd7 | Schnetz et al., 2010 |
| SetDB1, H3K9me3 | Bilodeau et al., 2009 |
| Dpy-30 | Jiang et al., 2011 |
| Atrx | Law et al., 2010 |
| YY1 | Mendenhall et al., 2010 |
| Prdm14 | Ma et al., 2010 |
